# Brain Peak Width of Skeletonized Mean Diffusivity (PSMD) and Cognitive Function in Later Life

**DOI:** 10.3389/fpsyt.2019.00524

**Published:** 2019-07-26

**Authors:** Ian J. Deary, Stuart J. Ritchie, Susana Muñoz Maniega, Simon R. Cox, Maria C. Valdés Hernández, Michelle Luciano, John M. Starr, Joanna M. Wardlaw, Mark E. Bastin

**Affiliations:** ^1^Department of Psychology, University of Edinburgh, Edinburgh, United Kingdom; ^2^Centre for Cognitive Ageing and Cognitive Epidemiology, University of Edinburgh, Edinburgh, United Kingdom; ^3^Social, Genetic and Developmental Psychiatry Research Centre, Institute of Psychiatry, Psychology and Neuroscience, King’s College London, London, United Kingdom; ^4^Brain Research Imaging Centre, Division of Neuroimaging Sciences, University of Edinburgh, Edinburgh, United Kingdom; ^5^Scottish Imaging Network, A Platform for Scientific Excellence (SINAPSE), University of Edinburgh, Edinburgh, United Kingdom; ^6^Edinburgh Dementia Research Centre, Dementia Research Institute, University of Edinburgh, Edinburgh, United Kingdom; ^7^Alzheimer Scotland Dementia Research Centre, University of Edinburgh, Edinburgh, United Kingdom

**Keywords:** aging, cognition, processing speed, structural MRI, diffusion MRI, white matter, PSMD

## Abstract

It is suggested that the brain’s peak width of skeletonized water mean diffusivity (PSMD) is a neuro-biomarker of processing speed, an important aspect of cognitive aging. We tested whether PSMD is more strongly correlated with processing speed than with other cognitive domains, and more strongly than other structural brain MRI indices. Participants were 731 Lothian Birth Cohort 1936 members, mean age = 73 years (SD = 0.7); analytical sample was 656–680. Cognitive domains tested were as follows: processing speed (5 tests), visuospatial (3), memory (3), and verbal (3). Brain-imaging variables included PSMD, white matter diffusion parameters, hyperintensity volumes, gray and white matter volumes, and perivascular spaces. PSMD was significantly associated with processing speed (−0.27), visuospatial ability (−0.23), memory ability (−0.17), and general cognitive ability (−0.25); comparable correlations were found with other brain-imaging measures. In a multivariable model with the other imaging variables, PSMD provided independent prediction of visuospatial ability and general cognitive ability. This incremental prediction, coupled with its ease to compute and possibly better tractability, might make PSMD a useful brain biomarker in studies of cognitive aging.

## Introduction

Cognitive functions such as processing speed, visuospatial reasoning, and some aspects of memory decline, on average, as people grow older, with deleterious effects on people’s quality of life (1–3). Processing speed has a special place among the cognitive domains; it has been suggested as a foundation for long-standing differences in, and aging of, other cognitive domains (4–9). With regard to understanding of cognitive aging, detailed attention has been advocated in the study of the psychometric aspects of processing speed and its potential neurobiological correlates (10). Therefore, understanding the neurobiological foundations of people’s differences in, and aging of, processing speed and other cognitive domains is a research priority (5, 11).

Even among those with no overt disease, brain-imaging-derived biomarkers of brain deterioration correlate with performance on cognitive tests. Such markers include cerebral tissue volumes and brain atrophy, and indicators of white matter health and so-called cerebral small vessel disease (12–17). Cerebral small vessel disease is an important contributor to vascular-based cognitive deterioration (18). Recognizing that, a brain imaging marker named “peak width of skeletonized [water] mean diffusivity” (PSMD) was developed and studied with respect to its association with processing speed (19). PSMD is based on skeletonization and histogram analysis of diffusion tensor magnetic resonance imaging (DT-MRI) data. PSMD was examined in comparison with other brain imaging markers in patients with inherited and sporadic cerebral small vessel disease, and in patients with Alzheimer’s disease and healthy controls (19). PSMD was reported to be associated with processing speed in all samples. The authors reported that it correlated with speed more strongly than other brain imaging markers such as brain volume, volume of white matter hyperintensities, and volume of lacunes.

However, although a special association between PSMD and the cognitive domain of processing speed was emphasized by Baykara et al. (19), no other cognitive domains were examined in their study. The Trail Making Test—measured in most of their samples—is associated with general fluid cognitive ability as well as processing speed (20–22). Therefore, PSMD might correlate with cognitive abilities more generally. As Schmidt (23) explained, all cognitive domains correlate positively together, and, therefore, associations with any one domain might represent an association with general cognitive function rather than, or in addition to, a domain-specific relationship. Before concluding that PSMD is a special neuro-biomarker of the important cognitive domain of processing speed, formal tests of whether PSMD correlates more strongly with processing speed, general cognitive ability, or other cognitive domains are needed. We sought to accomplish this in the present study.

Our present study includes experimental and psychophysical measures of processing speed in addition to more widely used paper-and-pencil assessments of speed. The former, arguably, rely less on acculturalized learning than do the paper-and-pencil-tests, and they are reckoned to be purer measures of speed (6, 24). Therefore, our study affords the examination of relatively specific tests of processing speed. Correlations of the different processing speed tests with PSMD are compared with those from a wide variety of other cognitive measures tapping broad cognitive domains. We also compare PSMD–cognitive function correlations with other structural brain imaging biomarkers’ cognitive correlations to test whether its association with processing speed and other cognitive domains is especially strong. Here, we run these analyses in a narrow-age sample of mostly healthy, community-dwelling older people: the Lothian Birth Cohort 1936 (LBC1936).

## Materials and Methods

### Participants

All participants were members of the LBC1936 (25–27). This started (LBC1936, Wave 1) as a sample of 1,091 people who were recruited between 2004 and 2007 at about 70 years of age. All lived independently in the community, were generally healthy, and travelled to a clinical research facility for assessment. Most of them had taken part in the Scottish Mental Survey 1947 at age 11 years. At Wave 1, they undertook extensive cognitive, medical, biomarker, psycho-social, and other assessments. The assessments were repeated at Wave 2, about 3 years later at mean age 73 years, with the addition of a detailed structural brain MRI scan (28). Of the 886 who returned at Wave 2, over 700 agreed to undertake a brain scan. All variables described below were collected at Wave 2, unless otherwise noted. Ethical approval for the LBC1936 study came from the Multi-Centre Research Ethics Committee for Scotland (MREC/01/0/56; 07/MRE00/58) and the Lothian Research Ethics Committee (LREC/2003/2/29). All participants, who were volunteers and received no financial or other reward, completed a written consent form before any testing took place.

### Cognitive Tests

The cognitive test data used here are from Wave 2, at mean age 73 years. All participants had already taken the same cognitive test battery at age 70 years, ensuring that they were familiar with the tests.

#### Processing Speed

Because processing speed was the key cognitive variable mentioned in relation with PSMD (19), it is apposite that the LBC1936 has been assessed for processing speed in detail. There were five tests of processing speed: two were paper-and-pencil psychometric tests; two were experimental, reaction time tests; and one was a psychophysical test. The psychometric tests were from the Wechsler Adult Intelligence Test-III^UK^: Digit Symbol and Symbol Search (29). The reaction time tests were simple reaction time (8 practice trials, 20 test trials) and 4-choice reaction time (8 practice trials, 40 test trials), assessed on a stand-alone device that was used in the UK’s Health and Lifestyle Study (24). The psychophysical test was inspection time, assessed using a bespoke computer program (30). The inspection time task required the participant to indicate which of two briefly presented parallel vertical lines was longer; no speeded response was required, and only the correctness of the response was recorded. Stimuli were backward-masked. There were 150 trials, with 10 trials at each of 15 durations, ranging from 6 ms to 200 ms. Durations were presented at random, using a method of constant stimuli.

#### Visuospatial Ability

This was assessed using Matrix Reasoning and Block Design from the Wechsler Adult Intelligence Test-III^UK^ (29) and Spatial Span (total score of forward and backward) from the Wechsler Memory Scales-III^UK^ (29).

#### Verbal Memory

This was assessed using Verbal Paired Associates (total score) and Logical Memory (immediate and delayed total score) from the Wechsler Memory Scales-III^UK^ (29) and Backward Digit Span from the Wechsler Adult Intelligence Test-III^UK^ (29).

#### Crystallized Ability

This was assessed using the National Adult Reading Test (31), the Wechsler Test of Adult Reading (32), and a phonemic verbal fluency test (using the letters C, F, and L) (25).

### Demographic and Health-Related Variables

The participant’s own occupational social class was based on their most prestigious job prior to retirement and assessed on a six-point scale from manual to professional (33). Education was assessed as the number of years of full-time education. These were assessed at Wave 1. Smoking was assessed at interview and classed as never, ex-, or current. The Mini-Mental State Examination (34) was used as a screen for cognitive pathology and not used as part of the cognitive test battery. Participants reported if they had a history of hypertension, cardiovascular disease, or diabetes.

### Magnetic Resonance Imaging

All MRI data were acquired using a GE Signa Horizon HDxt 1.5 T clinical scanner (General Electric, Milwaukee, WI, USA) using a self-shielding gradient set with a maximum gradient strength of 33 mT/m and an 8-channel phased-array head coil. The full details of the imaging protocol can be found in the LBC1936 imaging protocol paper (28). Briefly, the DT-MRI examination consisted of seven T_2_-weighted (*b* = 0 s mm^−2^) and sets of diffusion-weighted (*b* = 1,000 s mm^−2^) single-shot spin-echo echo-planar (EP) volumes acquired with diffusion gradients applied in 64 non-collinear directions (35). Volumes were acquired in the axial plane, with a field of view of 256 × 256 mm, contiguous slice locations, and image matrix and slice thickness designed to give 2-mm isotropic voxels. The repetition and echo time for each EP volume were 16.5 s and 98 ms, respectively. DT-MRI data were converted from DICOM (http://dicom.nema.org) to NIfTI-1 (http://nifti.-nimh.nih.gov/nifti-1) format using the TractoR package (http://www.tractor-mri.org.uk) (36). FSL tools (http://www.fmrib.ox.ac.uk/fsl) (37) were then used to extract the brain, remove bulk motion and eddy-current-induced distortions by registering all subsequent volumes to the first T_2_-weighted EP volume (38), estimate the water diffusion tensor, and calculate parametric maps of mean diffusivity (MD) and fractional anisotropy (FA) from its eigenvalues using DTIFIT (39).

#### Peak Width of Skeletonized Water MD

Automatic calculation of PSMD followed the procedure described by Baykara et al. (19) using the freely available script they provided (http://www.psmd-marker.com). Briefly, the DT-MRI data were processed using the standard Tract-based Spatial Statistics (TBSS) (40) pipeline available in FSL with histogram analysis performed on the resulting white matter MD skeletons. First, all participants’ FA volumes were linearly and non-linearly registered to the standard space FMRIB 1-mm FA template. Second, a white matter skeleton was created from the mean of all registered FA volumes. This was achieved by searching for maximum FA values in directions perpendicular to the local tract direction in the mean FA volume. An FA threshold of 0.2 was applied to the mean FA skeleton to exclude predominantly non-white matter voxels. Third, MD volumes were projected onto the mean FA skeleton and further thresholded at an FA value of 0.3 to reduce CSF partial volume contamination using the skeleton mask provided by Baykara et al. (19). Finally, PSMD was calculated as the difference between the 95th and 5th percentiles of the voxel-based MD values within each subject’s MD skeleton.

#### Other Structural Brain Imaging Variables

The estimation of, respectively, normal-appearing gray and white matter volumes, brain atrophy (intra-cranial volume minus total brain volume), general FA and general MD derived from quantitative tractography, white matter hyperintensity (WMH) volume, and visually rated perivascular spaces (41) from LBC1936 Wave 2 have all been described previously, as, mostly, have their associations with cognitive functions (11, 42–48). They are included in the present paper in order to compare their cognitive associations alongside those of PSMD, not as new results in themselves. Briefly, general FA and general MD were calculated using confirmatory factor analyses that estimated a latent factor from the (respective) FA or MD values of each of 12 broad white matter tracts (see Ref. 45 for a list). Each model included residual correlations between the left and right versions of the bilateral tracts.

### Statistical Analysis

All analyses were run in the *lavaan* package for R (49). Scores on each multi-test cognitive domain (processing speed, visuospatial ability, verbal memory, and crystallized ability) were derived from separate confirmatory factor analyses assessed using structural equation models based on the analytic sample. Scores on general cognitive ability were derived from a hierarchical confirmatory factor analysis where the score on the general cognitive factor represented the shared variance among the four cognitive domains. Associations between brain imaging parameters and cognitive test scores were conducted using linear regression, with *p* values adjusted for the false discovery rate (FDR) (50) as indicated in results tables. To test whether the relation of each cognitive test or domain with PSMD was significantly different from its relation with the other brain-derived variables, we used Williams’s test for differences in dependent correlations (implemented using the *psych* package in R) (51); we treated the standardized regression betas as correlations for this purpose. For each test, we accounted for the dependency (correlation) between the brain variables. Before inclusion in the analysis, we adjusted all variables for sex and age (in days) at the time of testing/scanning by regressing them on these two covariates and saving the residuals.

As an additional multivariable analysis, we ran a set of structural equation models in which all eight of the brain measures were entered simultaneously as predictors of the latent cognitive scores. This allowed us to test whether PSMD was incrementally significant beyond the more conventionally studied micro- and macro-structural brain measures. For the general FA and MD factors, we extracted the factor scores to use as predictors, but still estimated the cognitive factors as latent variables. As with the previous analyses, all variables were corrected for sex and age. Note that we already reported a similar analysis in this sample (46). However, the previous analysis did not include PSMD, and its aim was to discover how much of the variance in cognitive domains could be accounted for with structural brain variables. In the present analysis, the aim was novel, i.e., to test whether PSMD had an incremental contribution in predicting cognitive variation, in a situation where several structural brain variables were included simultaneously.

Finally, a reviewer suggested that we run an additional analysis that controlled for educational attainment and depressive symptoms. We did so, using the participants’ age-73 total score on the depression subscale of the Hospital Anxiety and Depression Scale (HADS) (52) and their self-reported total years of formal, full-time education, reported at age 70, as additional covariates in the model. That is, these variables were included in the regressions along with age and sex from which we saved residuals for further analysis.

## Results

Summary demographic, medical, brain imaging, and cognitive descriptive results are shown in **Table 1**. The total number of subjects who agreed to brain imaging was 731 (388 men), and between 656 and 680 of them provided brain imaging data that were able to be used to compute variables for use in this study. Their mean age was 72.7 years (SD = 0.72). About 47% had a history of hypertension, 27% had a history of cardiovascular disease, and 10% had a history of diabetes. Further, up to 35% had Fazekas WMH scores of 2 or 3 in peri-ventricular or deep white matter, an elevated rate given their age (see Ref. 53). Their mean Mini-Mental State Examination score was 28.8 (SD = 1.4).

**Table 1:** Characteristics of the Lothian Birth Cohort 1936 study sample. Numbers are mean and SD or *N* and %.

Variable category	Variable	Mean (SD) or *N* (%)	Total *N*
Demographic characteristics	Age (years)	72.68 (0.72)	866
	Sex; male, female	388, 343	1,091
	Adult social class	3.49 (1.21)	1,091
	Education (years)	10.79 (1.14)	1,091
	Smoking (never, ex-, current)	343, 327, 61	866
	Mini-Mental State examination	28.76 (1.41)	865
Medical conditions	Hypertension (%)	362 (46.9%)	866
	Cardiovascular disease (%)	198 (27.1%)	866
	Diabetes (%)	77 (10.5%)	866
	Hypercholesterolemia (%)	356 (41%)	866
	Depression (mean HADS score)	2.62 (2.22)	865
Structural brain imaging measures	PSMD	3.17 × 10^−4^ (5.01 × 10^−5^)	672
	General FA	0.00 (1.00)	664
	General MD	0.00 (1.00)	664
	WMH volume (cm^3^)	12.23 (12.18)	656
	Gray matter volume (cm^3^)	472.43 (44.68)	657
	White matter volume (cm^3^)	476.89 (50.55)	657
	Perivascular spaces	3.51 (1.10)	680
Processing speed tests	Wechsler Digit Symbol	56.29 (12.42)	862
	Wechsler Symbol Search	24.62 (6.19)	862
	Simple reaction time (s)	0.27 (0.05)	865
	4-choice reaction time (s)	0.65 (0.09)	865
	Inspection time	111.37 (11.75)	838
Visuospatial ability	Wechsler Matrix Reasoning	13.36 (4.92)	863
	Wechsler Block Design	34.00 (10.07)	864
	Wechsler Spatial Span	14.75 (2.77)	861
Verbal memory	Wechsler Verbal Paired Associates	27.18 (9.57)	843
	Wechsler Logical Memory	74.53 (17.92)	864
	Wechsler Digit Span Backward	7.86 (2.32)	866
Crystallized ability	National Adult Reading Test	34.36 (8.16)	864
	Wechsler Test of Adult Reading	41.01 (6.96)	864
	Phonemic verbal fluency	43.22 (12.95)	865

General FA and MD were latent variables created using full-information maximum-likelihood estimation from 12 white matter tract measurements; the total N given is the highest possible n. HADS, Hospital Anxiety and Depression Scale (total depression score was used for this analysis).

The age- and sex-adjusted correlations between the brain imaging measures used in this study are shown in **Supplementary Table 1**. PSMD correlated—in terms of absolute standardized effect sizes—above 0.5 with general FA and MD and WMH volume, about 0.3 with atrophy and perivascular spaces, and below 0.2 with gray and white matter volumes.

For clarity in understanding the results reported below, all significant associations were in the direction indicating that healthier brains had better performance in processing speed and other cognitive domains. “Healthier brains” had lower PSMD, higher general FA, lower general MD, lower WMH volume, higher gray and white matter volumes, lower atrophy, and fewer perivascular spaces.

### PSMD and Other Brain Imaging Variables Versus Processing Speed

We first examined the associations between the five tests of processing speed and PSMD and the other brain imaging measures (**Table 2**). Higher PSMD (representing less healthy white matter) was significantly associated with worse scores on all five measures of processing speed; the absolute standardized betas were between 0.11 and 0.23 (mean = 0.17). Those with higher PSMD had lower scores on Digit Symbol, Symbol Search, and inspection time, and slower simple and 4-choice reaction times. Therefore, PSMD does correlate significantly, in the expected direction, with these five methodologically varied tests of processing speed.

**Table 2:** The association between structural brain imaging parameters and individual tests of processing speed.

	Wechsler Digit Symbol			Wechsler Symbol Search			Simple reaction time			4-choice reaction time			Inspection time		
	β	SE	*p*_adj_	β	SE	*p*_adj_	β	SE	*p*_adj_	β	SE	*p*_adj_	β	SE	*p*_adj_
PSMD	−.226	.037	1.01 × 10^−8^	−.155	.038	5.74 × 10^−5^	.108	.037	.003	.169	.037	1.04 × 10^−5^	−.200	.037	3.02 × 10^−7^
General FA	.188	.041	1.70 × 10^−5^	.119	.042	.005	−.147	.044	.001	−.189	.042	1.70 × 10^−5^	.172	.042	8.14 × 10^−5^
General MD	−.143*	.041	.003	−.092	.042	.028	.121	.043	.008	.113	.043	.010	−.162	.042	5.05 × 10^−4^
WMH volume	−.200	.037	5.55 × 10^−7^	−.164	.038	3.13 × 10^−5^	.044	.037	.234	.140	.037	2.12 × 10^−4^	−.172	.039	2.45 × 10^−5^
Gray matter volume	.259	.037	2.62 × 10^−11^	.257^†^	.037	3.60 × 10^−11^	−.047	.037	.204	−.164	.037	1.48 × 10^−5^	.147	.039	2.05 × 10^−4^
White matter volume	.292	.036	2.55 × 10^−14^	.257^†^	.037	3.30 × 10^−11^	−.119	.037	.001	−.215	.036	7.45 × 10^−9^	.174	.039	1.07 × 10^−5^
Atrophy	.268	.037	4.05 × 10^−12^	.215	.038	3.16 × 10^−8^	−.109	.037	.003	−.180	.037	1.42 × 10^−6^	.220	.038	3.16 × 10^−8^
Perivascular spaces	−.033*	.038	0.646	.005*	.039	.899	.059	.037	.276	.005*	.038	.899	−.093*	.039	.085

All variables are adjusted for sex and age on the day of testing or MRI scanning. p_adj_ values are FDR-adjusted within each brain variable (that is, by rows of this table). *Significantly (absolutely) lower than the β for PSMD; ^†^Significantly (absolutely) higher than the β for PSMD. Standardized regression coefficients are reported throughout.

Normal-appearing gray and white matter volumes and brain atrophy had similar associations to PSMD with the five processing speed measures (**Table 2**); 11 of their 15 betas were larger in effect size (though not significantly larger) than those of PSMD. Associations between WMH volume and the processing speed tests were similar to those of PSMD, though mostly slightly lower. General FA and MD had significant associations with all five of the processing speed measures, with similar betas to those between PSMD and speed measures, though the majority were slightly lower. Perivascular spaces had non-significant associations with the five processing speed measures, and all were notably lower than those with PSMD. We repeated these analyses, omitting participants with a Mini-Mental State Examination score below 24; the results were very similar (**Supplementary Table 2**).

We tested the effect sizes of the associations between PSMD and individual processing speed tests to find out formally whether they were significantly stronger or weaker than those between other brain imaging variables and the same test. **Table 2** shows these results. Apart from perivascular spaces, where PSMD always had stronger associations with processing speed measures, PSMD had significantly stronger associations than other brain variables in only 1 of 30 comparisons.

### PSMD and Other Brain Imaging Variables’ Associations With Four Cognitive Domains and General Cognitive Ability

Next, we examined the possibility that PSMD showed significantly stronger associations with processing speed than with other cognitive domains, and whether they were significantly stronger or weaker associations compared to other brain imaging variables. We ascertained the associations between PSMD and other brain imaging measures and the four cognitive domains (each of which comprised multiple cognitive tests) and general cognitive ability (the variance shared by the four cognitive domains) (**Table 3**). Higher PSMD was significantly associated, at similar effect sizes, with poorer processing speed (standardized beta = −0.27), visuospatial ability (−0.23), and general cognitive ability (−0.25). The association with verbal memory was significant but lower (−0.17), and the association with crystallized ability was small and non-significant (−0.07).

**Table 3:** The association between structural brain imaging parameters and domains of cognitive ability and general cognitive ability.

	Processing speed			Visuospatial ability			Verbal memory			Crystallized ability			General cognitive ability		
	β	SE	*p*_adj_	β	SE	*p*_adj_	β	SE	*p*_adj_	β	SE	*p*_adj_	β	SE	*p*_adj_
PSMD	−.273	.040	4.64 × 10^−11^	−.235	.042	3.66 × 10^−8^	−.168	.045	2.37 × 10^−4^	−.072	.040	.070	−.250	.041	2.57 × 10^−9^
General FA	.238	.046	1.07 × 10^−6^	.174*	.048	4.44 × 10^−4^	.116	.050	.021	.100	.043	.021	.204	.047	2.89 × 10^−5^
General MD	−.178*	.046	5.76 × 10^−4^	−.163*	.047	.002	−.117	.050	.025	.003*	.043	.951	−.148*	.047	.003
WMH volume	−.247	.042	1.39 × 10^−8^	−.132*	.044	.004	−.137	.046	.004	−.048	.040	.231	−.184*	.043	4.19 × 10^−5^
Gray matter volume	.315	.039	6.42 × 10^−16^	.310	.041	4.46 × 10^−14^	.201	.044	3.97 × 10^−6^	.253^†^	.037	7.12 × 10^−12^	.357^†^	.038	3.95 × 10^−20^
White matter volume	.364^†^	.037	3.94 × 10^−22^	.280	.042	1.64 × 10^−11^	.175	.045	8.82 × 10^−5^	.239^†^	.037	1.21 × 10^−10^	.348	.038	2.45 × 10^−19^
Atrophy	.331	.039	5.89 × 10^−22^	.277	.042	5.63 × 10^−11^	.200	.045	1.06 × 10^−5^	.169^†^	.038	1.33 × 10^−5^	.320	.039	9.88 × 10^−16^
Perivascular spaces	.038*	.043	.638	−.054*	.044	.638	−.008*	.045	.867	.043	.040	.638	−.020*	.043	.798

All variables are adjusted for sex and age on the day of testing or MRI scanning. p_adj_ values are FDR-adjusted within each brain variable (that is, by rows of this table). *Significantly (absolutely) lower than the β for PSMD; ^†^Significantly (absolutely) higher than the β for PSMD. Standardized regression coefficients are reported throughout.

For all five of the cognitive variables, normal-appearing gray and white matter volumes and brain atrophy had stronger associations than PSMD (**Table 3**). The associations of WMH volume were somewhat lower than those of PSMD, and all but the association with crystallized ability were significant. General FA and MD had significant associations with all cognitive domains, except between MD and crystallized ability, and the betas were mostly slightly lower than those between PSMD and cognitive domains. Perivascular spaces had no significant associations with any cognitive domain.

We repeated these analyses, omitting participants with a Mini-Mental State Examination score below 24; the results were very similar (**Supplementary Table 3**). Additional analyses show the associations between the various structural brain imaging measures and the individual tests from the cognitive domains of Visuospatial ability, Memory, and Crystallized ability (**Supplementary Tables 4**, **5**, and **6**, respectively).

We tested the effect sizes of the associations between PSMD and each cognitive domain and general cognitive ability to find out formally whether they were significantly stronger or weaker than those between other brain imaging variables and the same cognitive variable. **Table 3** shows these results. There were 35 comparisons: in 11 of these, PSMD had stronger associations with cognitive domains and general cognitive ability, and in five of these, the PSMD associations were significantly weaker. For processing speed, PSMD was a significantly stronger associate than general MD and perivascular spaces, and significantly weaker than white matter volume. Therefore, although PSMD does correlate significantly, in the expected direction, with these cognitive measures, it did not exhibit the largest association among all brain measures in any cognitive domain.

### Multivariable Models to Test PSMD’s Incremental Contribution to Predicting Variance in Cognitive Domains and General Cognitive Ability

The results of the multivariable models, where all brain variables were entered together to predict variance in cognitive domain scores and general cognitive ability, are shown in **Table 4**. In these models, PSMD had a statistically significant association with visuospatial ability and with the general cognitive ability factor, but not with processing speed, verbal memory, or crystallized ability. No one brain measure consistently emerged as a statistically significant predictor for each cognitive ability. Gray matter volume, white matter volume, and atrophy were all independently significant alongside PSMD in predicting general cognitive ability. The general FA and MD variables were not independently significant in any of the models, indicating that variation in the cognitive abilities was better measured by either the macrostructural indices (except perivascular spaces, which were also not significant in any model), or by PSMD.

**Table 4:** Multivariable models predicting latent cognitive factors from all brain variables, entered simultaneously.

	Processing speed			Visuospatial ability			Verbal memory			Crystallized ability			General cognitive ability		
	β	SE	*p*_adj_	β	SE	*p*_adj_	β	SE	*p*_adj_	β	SE	*p*_adj_	β	SE	*p*_adj_
PSMD	0.093	0.060	0.240	**−0.184**	**0.063**	**0.024**	−0.095	0.068	0.416	−0.043	0.058	0.525	**−0.146**	**0.060**	**0.032**
General FA	−0.010	0.051	0.969	−0.066	0.055	0.365	−0.045	0.058	0.534	0.037	0.050	0.525	−0.026	0.052	0.813
General MD	0.002	0.052	0.969	−0.053	0.055	0.447	−0.043	0.059	0.534	0.067	0.051	0.470	−0.008	0.052	0.880
WMH volume	**0.140**	**0.053**	**0.021**	0.034	0.057	0.548	−0.076	0.060	0.416	−0.008	0.052	0.872	−0.062	0.054	0.406
Gray matter volume	−0.035	0.059	0.788	**0.173**	**0.062**	**0.024**	0.099	0.069	0.416	0.125	0.057	0.112	**0.149**	**0.059**	**0.032**
White matter volume	**−0.223**	**0.055**	**1.83 × 10** **^−4^**	0.120	0.059	0.084	0.047	0.064	0.534	0.132	0.053	0.104	**0.170**	**0.056**	**0.008**
Atrophy	**−0.220**	**0.048**	**3.69 × 10** **^−5^**	0.122	0.052	0.051	0.124	0.056	0.224	0.056	0.047	0.470	**0.171**	**0.049**	**0.004**
Perivascular spaces	−0.023	0.042	0.788	−0.030	0.045	0.548	0.028	0.048	0.552	0.032	0.042	0.525	0.016	0.043	0.816
*R* ^2^	0.229			0.159			0.082			0.081			0.214		

Values in bold had significant p_adj_ values. p_adj_ values were FDR-adjusted within each multivariable model (i.e., within columns of this table).

### Analyses With Additional Covariates

As suggested by a referee on an earlier version of this paper, we re-ran the main analyses with the additional controls of educational attainment and depressive symptoms (that is, alongside age and sex). We report the relevant results in **Supplementary Tables 7**, **8**, and **9** (which are the equivalents of **Tables 2**, **3**, and **4** in this main article, respectively). The addition of these covariates made little substantive difference to the results: effect sizes were changed at the second or third decimal place. In the multivariable analysis, some of the effect sizes were made slightly larger, though broadly the picture remained similar to that described above for the main analyses.

## Discussion

In this large sample of community-dwelling older people with a narrow age range around 73 years, we extended the previously reported (19) link between PSMD and processing speed to include necessary comparisons with a variety of more specific processing speed tests, other correlated cognitive domains, and other brain measures. In the present study, higher PSMD correlated significantly with poorer performance on five methodologically diverse tests of processing speed: two were psychometric, paper-and-pencil tests; two were experimental, reaction time tests; and one was a psychophysical test, requiring no fast motor response. Higher PSMD correlated significantly with poorer performance on the latent cognitive domain of processing speed, composed of tests of all three types. However, PSMD was not the exclusive or strongest associate of processing speed: normal-appearing gray and white matter volumes and brain atrophy were often slightly stronger, and WMH volume and tractography-based general FA and MD had slightly lower, but still significant associations. Moreover, PSMD did not have an especially strong association with processing speed compared with other cognitive domains; it correlated at similar levels with visuospatial ability and general cognitive ability, though less strongly with verbal memory and crystallized ability. In multivariable analyses, PSMD contributed predictive power, incremental to that of other structural brain imaging variables, to visuospatial ability and general cognitive ability.

If PSMD’s association with processing speed had been higher than those of other structural brain indices, and/or if PSMD has been especially strong in associating with the domain of processing speed by comparison with other cognitive domains, then there would have been strong evidence for arguing that PSMD stands out among brain imaging parameters in being especially associated with this important cognitive domain. Rather, the visuospatial cognitive domain and general cognitive ability were highlighted as having a heightened relationship with PSMD; that is, PSMD explained additional variance in these variables that was independent of the other brain imaging predictors. Because of its ease of computation and tractability, PSMD might be preferred over imaging measures that are burdensome to compute and which are more general properties of the brain. However, global measures of GM and WM volume can be obtained from a T1 alone, and their automated measurement can also be undertaken in an automated fashion. That is, both PSMD’s convenience and link to more specific brain biology might be used to argue in its favor; the average magnitude of water molecular diffusion in the center of common white matter pathways provides information on individual variations in white matter microstructure and the specific methods reduce the likelihood of influences of partial volume effects, rather than simply “how much” of a tissue type an individual possesses. Furthermore, code for performing PSMD is freely available and based on the commonly used TBSS pipeline, making it an accessible biomarker of white matter structure.

The similar association of PSMD with other cognitive domains might not necessarily detract from the claim that it is a marker of processing speed. This is because processing speed has long been seen as somewhat special among the cognitive domains. It has been argued that faster processing speed might provide a foundation for better cognitive performance in higher cognitive domains and for less cognitive decline in them (4, 8, 9). This is because the tests of processing speed are arguably simpler than those of other cognitive domains, often being referred to as elementary cognitive tests. Thus, if links to underlying biological processes are clearer for PSMD compared with other structural brain markers, and for processing speed compared with other cognitive domains, then it may be argued that PSMD is *primus inter* (apparent) *pares* for the brain indices examined here.

The study has the strengths of testing a large and age- and culturally homogeneous sample, all on the same brain scanner. The homogeneity of the sample is advantageous in that it means that there is not a “culture” variable within the sample that would need to be taken into account, with lowering of power, and as a possible source of different sizes of association. Processing speed was tested thoroughly, at three levels of description (psychometric, experimental, and psychophysical). Other important cognitive domains that show age-related mean decline—visuospatial reasoning and memory—and crystallized ability were assessed using multiple, well-validated tests. Also, recognizing that all cognitive domains correlate strongly, a general cognitive ability variable, capturing their shared variance, was included. The study has some limitations. The homogeneity of age and cultural background means that we are not able to generalize beyond those. Neither can we generalize beyond their status as relatively healthy, community-dwelling individuals, though we note that markers of small vessel disease indicated some pathology exceeding the expectations for their age. It is possible that samples with a broader range of brain pathology might show stronger brain imaging–cognitive domain associations, and possibly a pattern that brings out more of PSMD’s distinctiveness. For example, PSMD was associated with general cognitive ability and executive function in patients with WMLs but not in healthy controls (54). The original PSMD-validation study (19) had the advantage of multiple clinical and healthy groups, but its limitations included that most of its samples did not have a relatively specific test of processing speed and there were insufficient tests of other cognitive domains.

The present study assessed the usefulness of PSMD as a biomarker of cognitive function in older age. We found the predicted association with processing speed, but it was not exclusive or special with respect to other structural brain indices, or with respect to other cognitive domains. To sum up, we found that a) PSMD did not show especially high associations with processing speed when compared with other cognitive domains and b) other brain imaging variables correlated as highly or higher with cognitive variables—including processing speed—as did PSMD. However, those are not the only considerations. In the Introduction, we referred to the special status that some researchers consider processing speed to have with respect to its being a foundation for individual differences in cognitive ability and cognitive aging. We also pointed out that PSMD might have tractability, i.e., that it might be a more specific brain biomarker than other imaging measures. Therefore, PSMD’s specificity and processing speed’s possible special status among cognitive domains make the PSMD–processing speed association worth exploring and explaining in a wider range of clinical and non-clinical populations. For instance, there is recent evidence of PSMD’s association with speed tests in multiple sclerosis patients (55). Future research should also seek to clarify the nature of any distinctive relationships between PSMD with visuospatial and general cognitive abilities.

## Data Availability

The datasets generated for this study are available on request to the corresponding author.

## Ethics Statement

All subjects gave written informed consent in accordance with the Declaration of Helsinki. The protocol was approved by the Multi-Centre Research Ethics Committee for Scotland (MREC/01/0/56; 07/MRE00/58) and the Lothian Research Ethics Committee (LREC/2003/2/29).

## Author Contributions

ID drafted the manuscript and conceived the study aims; SR performed statistical analysis in discussion with ID; SM, SC, and MH processed the imaging data; ID, JS, JW, and MB supervised the larger cohort study. All authors commented on/edited the manuscript draft.

## Conflict of Interest Statement

The authors declare that the research was conducted in the absence of any commercial or financial relationships that could be construed as a potential conflict of interest.
