# Supplementary material for: Brain Peak Width of Skeletonized Mean Diffusivity (PSMD) and Cognitive Function in Later Life
Source: Front Psychiatry. 2019 Jul 26;10:524. doi: 10.3389/fpsyt.2019.00524 (PMC6676305; doi:10.3389/fpsyt.2019.00524)
Supplement: Supplementary file 1 [file DataSheet_1.docx]

**Supplementary Table 1**

The associations (Pearson’s *r*) between structural brain imaging parameters used in the study.

|  | PSMD | General FA | General MD | WMH volume | Grey matter volume | White matter volume | Atrophy |
| --- | --- | --- | --- | --- | --- | --- | --- |
| PSMD | - |  |  |  |  |  |  |
| General FA | −.59 | - |  |  |  |  |  |
| General MD | .61 | −.61 | - |  |  |  |  |
| WMH volume | .57 | −.32 | .51 | - |  |  |  |
| Grey matter volume | −.10 | .20 | .04 | −.09 | - |  |  |
| White matter volume | −.16 | .18 | −.16 | −.26 | .66 | - |  |
| Atrophy | −.29 | .39 | −.09 | −.01 | .48 | .28 |  |
| Perivascular spaces | .30 | −.28 | .33 | .28 | .09 | .03 | .02 |

Note. All associations are adjusted for sex and age at time of testing.

**Supplementary Table 2**

The association between structural brain imaging parameters and individual tests of processing speed, excluding participants with Mini-Mental State Examination score < 24.

|  | Wechsler Digit Symbol | | | Wechsler Symbol Search | | | Simple reaction time | | | 4-choice reaction time | | | Inspection time | | |
| --- | --- | --- | --- | --- | --- | --- | --- | --- | --- | --- | --- | --- | --- | --- | --- |
|  | *β* | SE | *p*_adj_ | *β* | SE | *p*_adj_ | *β* | SE | *p*_adj_ | *β* | SE | *p*_adj_ | *β* | SE | *p*_adj_ |
| PSMD | −.227 | .037 | 9.27×10^-09^ | −.158 | .038 | 4.49×10^-05^ | .113 | .038 | 3.21×10^-03^ | .169 | .037 | 9.54×10^-06^ | −.201 | .038 | 3.13×10^-07^ |
| General FA | .190 | .041 | 1.61×10^-05^ | .121 | .042 | .004 | −.142 | .042 | .001 | −.189 | .042 | 1.61×10^-05^ | .172 | .042 | 7.97×10^-05^ |
| General MD | −.144* | .042 | .003 | −.096 | .042 | .021 | .119 | .042 | .007 | .114 | .043 | .009 | −.165 | .042 | 3.89×10^-04^ |
| WMH volume | −.205 | .038 | 3.64×10^-07^ | −.170 | .039 | 1.95×10^-05^ | .047 | .039 | .223 | .143 | .037 | 1.60×10^-04^ | −.174 | .039 | 1.95×10^-05^ |
| Grey matter volume | .258 | .037 | 3.93×10^-11^ | .253 | .038 | 1.21×10^-10^ | −.050 | .039 | .198 | −.164 | .037 | 1.76×10^-05^ | .143 | .039 | 3.32×10^-04^ |
| White matter volume | .294 | .039 | 2.39×10^-14^ | .255† | .038 | 7.93×10^-11^ | −.120 | .037 | .002 | −.212 | .037 | 1.61×10^-08^ | .172 | .039 | 1.62×10^-05^ |
| Atrophy | .263 | .039 | 1.56×10^-11^ | .203 | .038 | 2.63×10^-07^ | −.114 | .038 | .003 | −.176 | .037 | 2.89×10^-06^ | .213 | .039 | 1.16×10^-07^ |
| Perivascular spaces | −.032* | .038 | .671 | .003* | .039 | .934 | .063 | .038 | .241 | .005* | .038 | .934 | −.095* | .039 | .076 |

Note. All variables are adjusted for sex and age on the day of testing or MRI scanning. *p*_adj_-values are FDR-adjusted within each brain variable (that is, by rows of this table). * = significantly (absolutely) lower than the *β* for PSMD; † = significantly (absolutely) higher than the *β* for PSMD. Standardised regression coefficients are reported throughout.

**Supplementary Table 3**

The association between structural brain imaging parameters and domains of cognitive ability and general cognitive ability, excluding participants with Mini-Mental State Examination score < 24.

|  | Processing speed | | | Visuospatial ability | | | Verbal memory | | | Crystallised ability | | | General cognitive ability | | |
| --- | --- | --- | --- | --- | --- | --- | --- | --- | --- | --- | --- | --- | --- | --- | --- |
|  | *β* | SE | *p*_adj_ | *β* | SE | *p*_adj_ | *β* | SE | *p*_adj_ | *β* | SE | *p*_adj_ | *β* | SE | *p*_adj_ |
| PSMD | −.275 | .040 | 3.21×10^-11^ | −.241 | .042 | 1.95×10^-08^ | −.174 | .045 | 1.45×10^-04^ | −.083 | .040 | .038 | −.258 | .041 | 8.41×10^-10^ |
| General FA | .239 | .046 | 7.86×10^-07^ | .175 | .048 | 4.58×10^-04^ | .121 | .050 | .016 | .111 | .044 | .014 | .210 | .047 | 1.64×10^-15^ |
| General MD | −.181 | .046 | 4.23×10^-04^ | −.168 | .048 | .001 | −.125 | .050 | .017 | −.008* | .044 | .864 | −.154* | .047 | .002 |
| WMH volume | −.251 | .041 | 6.56×10^-09^ | −.139 | .044 | .002 | −.144 | .046 | .002 | −.058 | .041 | .153 | −.193* | .043 | 4.19×10^-05^ |
| Grey matter volume | .311 | .040 | 1.05×10^-14^ | .304 | .041 | 3.13×10^-13^ | .192 | .044 | 1.51×10^-05^ | .250† | .037 | 3.07×10^-11^ | .352† | .039 | 3.95×10^-20^ |
| White matter volume | .360 | .038 | 1.66×10^-20^ | .279 | .042 | 4.75×10^-11^ | .164 | .046 | 3.32×10^-04^ | .233† | .038 | 8.54×10^-10^ | .345 | .039 | 2.66×10^-19^ |
| Atrophy | .319 | .039 | 2.84×10^-15^ | .262 | .043 | 1.35×10^-09^ | .181 | .046 | 9.73×10^-05^ | .148 | .040 | 1.76×10^-04^ | .300 | .040 | 2.67×10^-13^ |
| Perivascular spaces | .039 | .043 | .671 | −.059 | .045 | .671 | −.012* | .045 | .797 | .030 | .040 | .671 | −.027* | .044 | .671 |

Note. All variables are adjusted for sex and age on the day of testing or MRI scanning. *p*_adj_-values are FDR-adjusted within each brain variable (that is, by rows of this table). * = significantly (absolutely) lower than the *β* for PSMD; † = significantly (absolutely) higher than the *β* for PSMD. Standardised regression coefficients are reported throughout.

**Supplementary Table 4**

The association between structural brain imaging parameters and individual tests of cognitive ability from the visuospatial domain.

|  | Wechsler Matrix Reasoning | | |  | Wechsler Block Design | | |  | Wechsler Spatial Span | | |  |
| --- | --- | --- | --- | --- | --- | --- | --- | --- | --- | --- | --- | --- |
|  | *β* | SE | *p*_adj_ |  | *β* | SE | *p*_adj_ |  | *β* | SE | *p*_adj_ |  |
| PSMD | -.16 | .04 | 6.21×10^-05^ |  | -.20 | .04 | 1.21×10^-06^ |  | -.11 | .04 | .006 |  |
| General FA | -.09* | .04 | .07 |  | .16 | .04 | 2.70×10^-04^ |  | .06 | .04 | .19 |  |
| General MD | .08* | .04 | .08 |  | -.15 | .06 | .001 |  | -.07 | .04 | .09 |  |
| WMH volume | -.09 | .04 | .03 |  | -.11* | .04 | .01 |  | -.05 | .04 | .17 |  |
| Grey matter volume | .17 | .04 | 1.34×10^-05^ |  | .25 | .04 | 2.35×10^-10^ |  | .21 | .04 | 1.11×10^-07^ |  |
| White matter volume | .17 | .04 | 1.81×10^-05^ |  | .23 | .04 | 1.40×10^-08^ |  | .17 | .04 | 1.81×10^-05^ |  |
| Atrophy | .16 | .04 | 5.30×10^-05^ |  | .22 | .04 | 4.50×10^-08^ |  | .19 | .04 | 1.10×10^-06^ |  |
| Perivascular spaces | -.02* | .04 | .65 |  | -.05* | .04 | .36 |  | -.05 | .04 | .36 |  |

Note. All associations are adjusted for sex and age at time of testing. *p*_adj_-values are FDR-adjusted within each brain variable (that is, by rows of this table). * = significantly (absolutely) lower than the *β* for PSMD; † = significantly (absolutely) higher than the *β* for PSMD. Standardised regression coefficients are reported throughout.

**Supplementary Table 5**

The association between structural brain imaging parameters and individual tests of cognitive ability from the memory domain.

|  | Wechsler Logical Memory | | |  | Wechsler Verbal Paired Associates | | |  | Wechsler Digit Span Backward | | |  |
| --- | --- | --- | --- | --- | --- | --- | --- | --- | --- | --- | --- | --- |
|  | *β* | SE | *p*_adj_ |  | *β* | SE | *p*_adj_ |  | *β* | SE | *p*_adj_ |  |
| PSMD | -.09 | .04 | .02 |  | -.14 | .04 | .001 |  | -.12 | .04 | .003 |  |
| General FA | .05 | .04 | .18 |  | .10 | .04 | .03 |  | .10 | .04 | .03 |  |
| General MD | -.05 | .04 | .28 |  | -.15 | .04 | .001 |  | -.05* | .04 | .28 |  |
| WMH volume | -.07 | .04 | .09 |  | -.14 | .04 | .001 |  | -.05 | .04 | .164 |  |
| Grey matter volume | .16 | .04 | 7.73×10^-05^ |  | .10 | .04 | 8.88×10^-03^ |  | .16 | .04 | 7.18×10^-05^ |  |
| White matter volume | .09 | .04 | .01 |  | .15 | .04 | 5.63×10^-04^ |  | .13 | .04 | 8.30×10^-04^ |  |
| Atrophy | .11 | .04 | .007 |  | .16 | .04 | 1.37×10^-04^ |  | .15 | .04 | 2.89×10^-04^ |  |
| Perivascular spaces | .01 | .04 | .66 |  | -.03* | .04 | .66 |  | -.02* | .04 | .66 |  |

Note. All associations are adjusted for sex and age at time of testing. *p*_adj_-values are FDR-adjusted within each brain variable (that is, by rows of this table). * = significantly (absolutely) lower than the *β* for PSMD; † = significantly (absolutely) higher than the *β* for PSMD. Standardised regression coefficients are reported throughout.

**Supplementary Table 6**

The association between structural brain imaging parameters and individual tests of cognitive ability from the crystallized domain.

|  | NART | | |  | WTAR | | |  | Phonemic verbal fluency | | |  |
| --- | --- | --- | --- | --- | --- | --- | --- | --- | --- | --- | --- | --- |
|  | *β* | SE | *p*_adj_ |  | *β* | SE | *p*_adj_ |  | *β* | SE | *p*_adj_ |  |
| PSMD | -.05 | .04 | .16 |  | -.07 | .04 | .08 |  | -.12 | .04 | .002 |  |
| General FA | .09 | .04 | .03 |  | .09 | .04 | .03 |  | .09 | .04 | .03 |  |
| General MD | -.005 | .04 | .90 |  | .01 | .04 | .90 |  | -.08 | .04 | .15 |  |
| WMH volume | -.03 | .04 | .48 |  | -.06 | .04 | .22 |  | -.09 | .04 | .07 |  |
| Grey matter volume | .24† | .04 | 9.32×10^-10^ |  | .24† | .04 | 1.10×10^-09^ |  | .15 | .04 | 1.45×10^-04^ |  |
| White matter volume | .22† | .04 | 1.31×10^-08^ |  | .23† | .04 | 1.08×10^-08^ |  | .20 | .04 | 3.08×10^-07^ |  |
| Atrophy | .14 | .04 | 2.98×10^-04^ |  | .17† | .04 | 3.09×10^-05^ |  | .15 | .04 | 2.08×10^-04^ |  |
| Perivascular spaces | .03 | .04 | .50 |  | .05 | .04 | .50 |  | .03 | .04 | .50 |  |

Note. All associations are adjusted for sex and age at time of testing. *p*_adj_-values are FDR-adjusted within each brain variable (that is, by rows of this table). * = significantly (absolutely) lower than the *β* for PSMD; † = significantly (absolutely) higher than the *β* for PSMD. Standardised regression coefficients are reported throughout.

**Supplementary Table** **7**

The association between structural brain imaging parameters and individual tests of processing speed, adjusted for age at testing/scanning, sex, years of education, and HADS depression subscore.

|  | Wechsler Digit Symbol | | | Wechsler Symbol Search | | | Simple reaction time | | | 4-choice reaction time | | | Inspection time | | |
| --- | --- | --- | --- | --- | --- | --- | --- | --- | --- | --- | --- | --- | --- | --- | --- |
|  | *β* | SE | *p*_adj_ | *β* | SE | *p*_adj_ | *β* | SE | *p*_adj_ | *β* | SE | *p*_adj_ | *β* | SE | *p*_adj_ |
| PSMD | −.246 | .037 | 3.24×10^-10^ | −.169 | .038 | 1.61×10^-05^ | .106 | .037 | .004 | .162 | .037 | 2.01×10^-05^ | −.207 | .038 | 1.32×10^-07^ |
| General FA | .188 | .041 | 2.99×10^-05^ | .116 | .042 | .006 | −.140 | .043 | .001 | −.177 | .042 | 6.79×10^-05^ | .169 | .042 | 9.30×10^-05^ |
| General MD | −.137 | .040 | 4.73×10^-04^ | −.100 | .042 | .017 | .119 | .043 | .010 | .108 | .043 | .015 | −.166 | .041 | 3.00×10^-04^ |
| WMH volume | −.214 | .037 | 6.45×10^-08^ | −.175 | .038 | 1.05×10^-05^ | .044 | .037 | .235 | .140 | .037 | 2.07×10^-04^ | −.177 | .039 | 1.05×10^-05^ |
| Grey matter volume | .224 | .037 | 6.40×10^-09^ | .230 | .037 | 3.40×10^-09^ | −.030 | .037 | .426 | −.142 | .037 | 2.16×10^-04^ | .128 | .039 | .001 |
| White matter volume | .253 | .036 | 7.26×10^-11^ | .224 | .038 | 1.31×10^-08^ | −.103 | .038 | .005 | −.197 | .037 | 1.75×10^-07^ | .153 | .039 | 1.13×10^-04^ |
| Atrophy | .243 | .037 | 5.28×10^-10^ | .195 | .038 | 6.83×10^-07^ | −.092 | .037 | .013 | −.153 | .037 | 4.65×10^-05^ | .210 | .039 | 1.84×10^-07^ |
| Perivascular spaces | −.041 | .038 | 0.474 | 8.07×10^-05^ | .038 | .998 | .005 | .037 | .263 | .005 | .038 | .998 | −.096 | .039 | .068 |

Note. *p*_adj_-values are FDR-adjusted within each brain variable (that is, by rows of this table). Standardised regression coefficients are reported throughout.

**Supplementary Table** **8**

The association between structural brain imaging parameters and domains of cognitive ability and general cognitive ability, adjusted for age at testing/scanning, sex, years of education, and HADS depression subscore.

|  | Processing speed | | | Visuospatial ability | | | Verbal memory | | | Crystallised ability | | | General cognitive ability | | |
| --- | --- | --- | --- | --- | --- | --- | --- | --- | --- | --- | --- | --- | --- | --- | --- |
|  | *Β* | SE | *p*_adj_ | *β* | SE | *p*_adj_ | *β* | SE | *p*_adj_ | *β* | SE | *p*_adj_ | *β* | SE | *p*_adj_ |
| PSMD | −.285 | .039 | 1.22×10^-12^ | −.282 | .042 | 4.41×10^-11^ | −.200 | .047 | 2.34×10^-05^ | −.142 | .039 | 2.87×10^-04^ | −.323 | .040 | 7.14×10^-15^ |
| General FA | .238 | .046 | 1.30×10^-06^ | .185 | .049 | 2.68×10^-04^ | .121 | .051 | .019 | .135 | .044 | 2.99×10^-03^ | .235 | .048 | 2.47×10^-06^ |
| General MD | −.188 | .046 | 1.24×10^-04^ | −.190 | .048 | 1.40×10^-04^ | −.141 | .052 | .008 | −.030 | .045 | .499 | −.196 | .048 | 1.24×10^-04^ |
| WMH volume | −.256 | .041 | 1.66×10^-09^ | −.153 | .046 | .001 | −.155 | .048 | .002 | −.077 | .040 | .056 | −.229 | .043 | 3.18×10^-07^ |
| Grey matter volume | .272 | .040 | 3.57×10^-11^ | .235 | .044 | 9.52×10^-11^ | .112 | .048 | 4.32×10^-04^ | .226 | .038 | 3.79×10^-09^ | .333 | .040 | 8.90×10^-16^ |
| White matter volume | .316 | .039 | 2.73×10^-15^ | .280 | .042 | 1.61×10^-07^ | .175 | .045 | .021 | .182 | .039 | 3.91×10^-06^ | .305 | .037 | 4.92×10^-13^ |
| Atrophy | .294 | .040 | 3.87×10^-13^ | .270 | .044 | 9.12×10^-10^ | .175 | .048 | 2.41×10^-04^ | .171 | .039 | 1.53×10^-05^ | .323 | .041 | 1.28×10^-14^ |
| Perivascular spaces | .044 | .043 | .580 | −.072 | .045 | .557 | −.017 | .047 | .712 | .029 | .040 | .580 | −.042 | .045 | .580 |

Note. *p*_adj_-values are FDR-adjusted within each brain variable (that is, by rows of this table). Standardised regression coefficients are reported throughout.

**Supplementary Table** **9**

Multivariable models predicting latent cognitive factors from all brain variables, entered simultaneously. Each brain and cognitive variable adjusted for age at testing/scanning, sex, years of education, and HADS depression subscore.

|  | Processing speed | | | Visuospatial ability | | | Verbal memory | | | Crystallised ability | | | General cognitive ability | | |
| --- | --- | --- | --- | --- | --- | --- | --- | --- | --- | --- | --- | --- | --- | --- | --- |
|  | *β* | SE | *p*_adj_ | *β* | SE | *p*_adj_ | *β* | SE | *p*_adj_ | *β* | SE | *p*_adj_ | *β* | SE | *p*_adj_ |
| PSMD | 0.125 | 0.061 | 0.080 | **-0.269** | **0.065** | **3.00×10^-05^** | -0.146 | 0.070 | 0.277 | -0.107 | 0.059 | 0.288 | **-0.229** | **0.062** | **1.91×10^-04^** |
| General FA | -0.002 | 0.053 | 0.969 | -0.087 | 0.058 | 0.210 | -0.049 | 0.061 | 0.555 | 0.062 | 0.051 | 0.450 | -0.034 | 0.055 | 0.613 |
| General MD | -0.016 | 0.053 | 0.874 | -0.043 | 0.058 | 0.607 | -0.042 | 0.062 | 0.575 | 0.078 | 0.052 | 0.363 | 0.003 | 0.055 | 0.959 |
| WMH volume | **0.153** | **0.053** | **0.011** | 0.034 | 0.059 | 0.640 | -0.059 | 0.062 | 0.554 | -0.033 | 0.053 | 0.532 | -0.078 | 0.056 | 0.258 |
| Grey matter volume | -0.037 | 0.060 | 0.719 | **0.194** | **0.064** | **0.005** | 0.117 | 0.072 | 0.277 | 0.156 | 0.058 | 0.056 | **0.174** | **0.061** | **0.011** |
| White matter volume | **-0.183** | **0.056** | **.004** | 0.045 | 0.061 | 0.005 | -0.030 | 0.067 | 0.650 | 0.034 | 0.055 | 0.532 | 0.090 | 0.058 | 0.242 |
| Atrophy | **-0.185** | **0.049** | **3.69×10^-05^** | 0.114 | 0.054 | 0.070 | 0.107 | 0.060 | 0.277 | 0.045 | 0.049 | 0.475 | **0.162** | **0.051** | **0.004** |
| Perivascular spaces | -0.060 | 0.044 | 0.283 | -0.022 | 0.048 | 0.655 | 0.047 | 0.051 | 0.554 | 0.043 | 0.043 | 0.475 | 0.041 | 0.046 | 0.501 |
| *R*^2^ | 0.193 | | | 0.164 | | | 0.072 | | | 0.074 | | | 0.221 | | |

*Note:* Values in bold had significant *p*_adj_-values. *p*_adj_-values were FDR-adjusted within each multivariate model (i.e. within columns of this table).
